# Spatial-temporal variation characteristics and evolution of the global industrial robot trade: A complex network analysis

**DOI:** 10.1371/journal.pone.0222785

**Published:** 2019-09-26

**Authors:** Yaya Li, Yongtao Peng, Jianqiang Luo, Yihan Cheng, Eleonora Veglianti

**Affiliations:** 1 School of Finance & Economics, Jiangsu University, Zhenjiang, Jiangsu, P.R. China; 2 School of Management, Jiangsu University, Zhenjiang, Jiangsu, P.R. China; 3 Department of Economics, University of Uninettuno, Roma, Italy; Harbin Institute of Technology, CHINA

## Abstract

Industrial robots are a strategic future technology and an important part of the development of artificial intelligence, and they are a necessary means for the intelligent transformation of manufacturing industry. Based on global industrial robot trade data from 1998 to 2017, this paper applies the dynamic complex network analysis method to reveal the spatial and temporal variation characteristics and trade status evolution of the global industrial robot trade network. The results show that the global industrial robot network density has steadily increased, and the industrial robot trade has been characterized by ‘diversification’. The number of major industrial robot exporters in the world is increasing, and the import market is increasingly diversified. The export market structure is relatively tight, the centrality of the global industrial robot trade network shows a downward trend, and the dissimilarity of the ‘core-edge’ clusters decreases year by year. The trade status of ‘catch-up’ countries represented by China has rapidly increased. However, Japan, Germany, and Italy are still in the central position of the industrial robot trade. Moreover, trade of the ‘catch-up’ countries’ is dominated by imports, and exports of industrial robot products are insufficient. Finally, policy suggestions are provided according to the results.

## Introduction

Industrial robots are a strategic future technology that represent, an important part of the development of artificial intelligence and a necessary means for the intelligent transformation of the manufacturing industry. According to the latest research from the International Federation of Robotics (IFR), the average global sales of industrial robots increased by 19% from 2012 to 2017, with 30% growth in 2017, totaling 381,335 units; therefore, the global demand for industrial robots has sharply increased [1]. China has been the world's largest industrial robot market since 2014. In the past 20 years, the following question has emerged: what is the global industrial robot trade pattern, and what are its evolutionary characteristics? To the best of our knowledge, studies have not yet answered these two questions. This study is the first to investigate the characteristics and evolution of the global industrial robot trade. Accordingly, this study offers two main contributions. First, it analyzes the spatial and temporal variation characteristics of the global industrial robot trade network. Second, it reveals the trade status evolution of global industrial robot trade network, especially pushed by ‘catch-up’ countries.

As industrial robots become the core elements of smart manufacturing, academic circles are paying increasingly more attention. In terms of the impact of industrial robots on employment, Graetz & Michaels pointed out that industrial robots have greater substitutability for general employment in labor-intensive enterprises [2], but they complement highly skilled labor and increase the demand for skilled personnel [3]. In terms of the industrial robot industry’s development, few scholars have studied the technological development of industrial robots based on patent data and provided technology intelligence analysis [4]. Many recent studies have focused on open innovation and convergence innovation [5–6]. Some scholars have proposed that robotics is an emerging technology with cross-disciplinary convergence [7–9], and technological breakthroughs and business model innovations are crucial to the development of the robotics industry [10]. In the field of the industrial robot trade, few studies believed that the application of industrial robot technology has increased the import and export volumes after examining the effects of robotization on trade patterns, wages and welfare [11]. However, few studies attempted to explore the characteristics and evolution of the global industrial robot trade network.

In addition, identification of the trade network structure and evolutionary characteristics based on complex network analysis has been widely used by scholars [12–16]. Thus far, investigations have been confined to the Belt and Road trade network [17–18], the global resource products trade network (such as fossil energy, crude oil, natural gas and rare earths) [13,19–22], and the international agro-food trade network [23]. Few researchers have examined the characteristics of trade networks in the manufacturing sector [24–25]. The above studies discuss the evolutionary characteristics of some industries that play an important role in highlighting our research. For example, international crude oil trade is evolving into a stable, ordered and integrated system [19], the international rare earth trade network is dispersed and unstable [22], three energy-specific networks, namely, coal, oil, and natural gas, display scale-free characteristics [13], trade regionalization is still high in the electronic industry, geographical proximity still plays a role in facilitating international trade [25], etc.

The purpose of this paper is to reveal the structural characteristics and evolution of the global industrial robot trade network by considering the network analysis method that has the following advantages. First, with respect to the dynamic topological analysis of the trade network, the imbalance of the global trade supply and demand accelerates the internationalization of trade, and the relationship between the supply and demand of commodities in each country of the network can reflect the spatial and temporal characteristics of the commodity trade network. Further, investigating the network density, degree, closeness and betweenness centrality can allow one to assess the statuses of the network nodes in the whole network. Therefore, it can effectively depict the strength of the trade relations among countries in the global trade network and better explain the global trade network [13].

Although considerable research has been devoted to the global commodity trade network, relatively less attention has been paid to industrial robots. This study is designed to depict the network topological structure of the global industrial robot trade and reveal the spatial-temporal differentiation and evolution of the global industrial robot trade. The remainder of this paper is divided into five sections: section 2 presents the network construction and describes the indicators, section 3 depicts the spatial-temporal differentiation of the global industrial robot trade, section 4 investigates the evolution of the global industrial robot trade, and section 5 concludes the paper.

## Construction and indicators of the global industrial robot trade network

### Construction of the network

**Fig 1:**
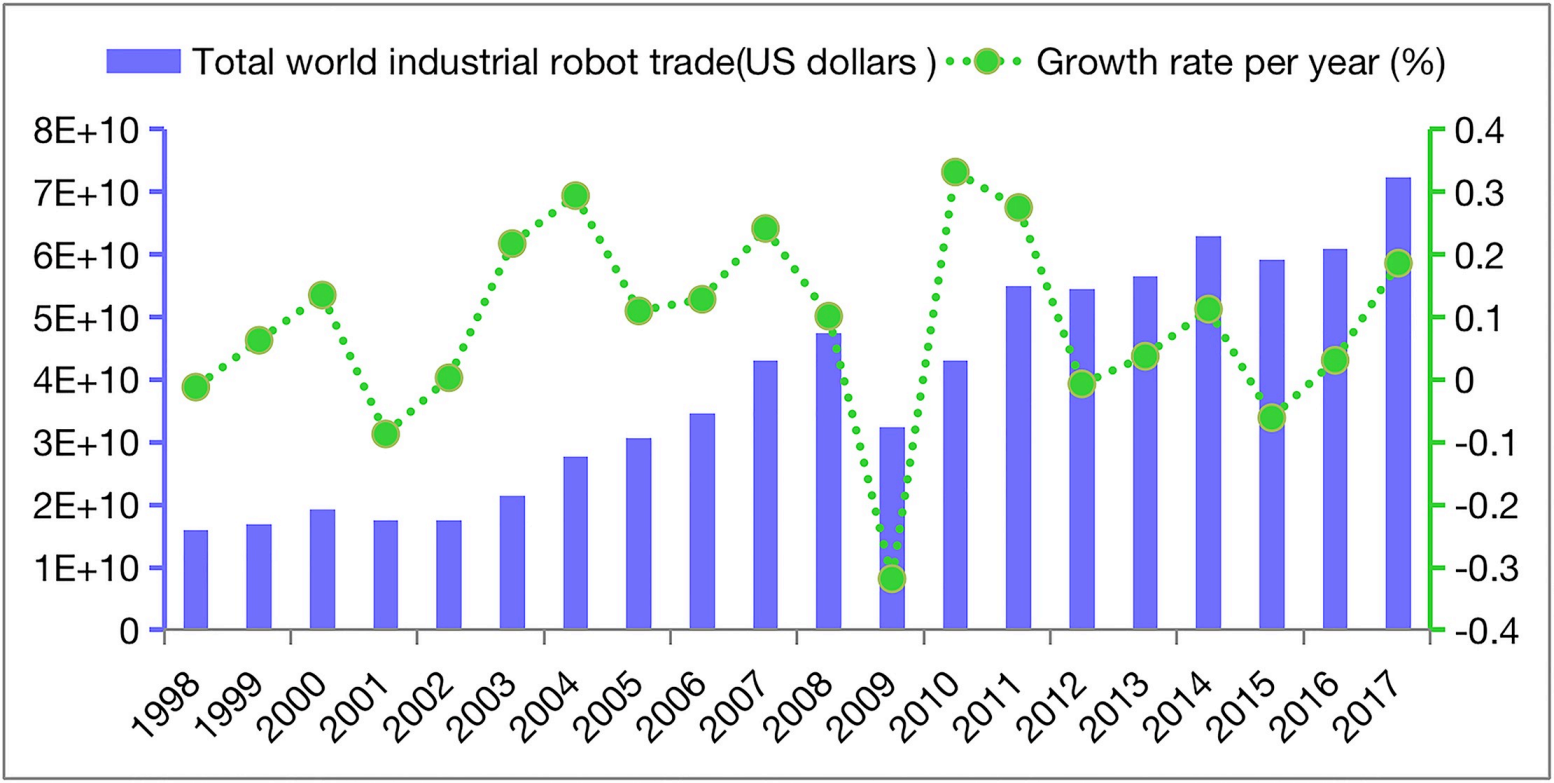
Total value and growth rate of the international industrial robot trade from 1998–2017.

Complex network analysis is a kind of network analysis method developed by sociologists based on mathematical theory. At present, the complex network analysis method has been applied to social and economic fields [13, 26–28]. The global trade network is a network that is used to describe the trade links between node countries—i.e., the links between node countries represent the import and export trade relations between them, as well as the structural and evolutionary characteristics of the global trade network by measuring the density, centrality, block model and other indicators [29,14]. This paper constructs a directed weighted global industrial robot trade network (GTN). The GTN was built using the set *G* = (*V*,*W*) in which the nodes *v* = {*v*_*i*_:*i* = 1,2,3..,*n*} represent the trading countries. The trading relationship between *v*_*i*_ and *v*_*j*_ is denoted by *a*_*ij*_. If country *v*_*i*_ exports industrial robots to country *v*_*j*_, a link from *v*_*i*_ to *v*_*j*_ is drawn and *a*_*ij*_ = 1; otherwise, no link is drawn, and *a*_*ij*_ = 0. *w* = {*w*_*ij*_} represents the weights of the edges in the network. Namely, the nodes are the countries, and the edges are the trade volume. The directions of the edges correspond to the directions of the industrial robot trade flow. The exports and imports represent the trade flows out of and into a country, respectively. According to the above method, this paper collects import and export trade data between 58 major industrial robot trading economies (see S1 Appendix for a detailed list) from the United Nations Commodity Trade Statistics Database (Uncomtrade). (The used HS codes are 842489, 842890, 847950, 848640, 851521, 851531, and 851580.) The time interval of the data is chosen as 1998 to 2017. Since the international industrial robot trade mushroomed after 1998, the international trade value of industrial robots in 2017 was quadruple that in 1998, as shown in Fig 1. In addition, both the trade value and growth rate declined in 2009 due to the lag effect of financial crisis in the whole world. Furthermore, 2009 is not considered a typical sample year to demonstrate the evolution of global industrial robot trade since the economy soon recovered in 2010.

### Indicators of network analysis

The **network density** index, which has been proved to be effective for measuring the density of networks, here depicts the extent of the connections among the nodes in the GTN. It equals the ‘total number of relationships that actually exist divided by the ‘maximum number of relationships that theoretically exist potentially’ [30]. In a way, the network density in this paper represents the extent of the network’s evolution and the industrial robot trade’s development. The larger this value is, the closer the relationships are among all of the countries. The definition of the network density is given by Eq (1), where *m* is the number of actual relationships in a network, and the number of nodes is *n*.

$$D=\frac{2m}{n(n-1)}$$ (1)

**Degree** refers to the number of direct trade relationships that a country has, and it reflects the range of the country’s direct impact. The out-degree is the number of export links that a country has with other countries, and the in-degree is the number of import links. A higher out-degree or in-degree indicates a higher direct impact among other countries [31]. These values are computed according to the following equations:

$$k_{i}^{out}(t)=\sum_{j=1}^{n}d_{ij}(t)$$ (2)

$$k_{i}^{in}(t)=\sum_{j=1}^{n}d_{ji}(t)$$ (3)

Here, if country *i* exports industrial robots to country *j* during year *t*, a link from *i* to *j* is drawn, and *d*_*ij*_(*t*) = 1. Otherwise, no link is drawn, and *d*_*ij*_(*t*) = 0. The out degree $k_{i}^{out}(t)$ of country i in year t is the sum of the *d*_*ij*_(*t*), and the in degree $k_{i}^{in}(t)$ of country *i* in the year *t* is the sum of the *d*_*ji*_(*t*).

The **closeness centrality** index reflects to what degree a country stands at the central position of the network. The more central a country is, the lower is its total distance from all the other nodes. The closeness centrality of node *i* is given by Eq (4):

$$CC(i)=\frac{1}{\sum_{i\neq j}d(i,j)}$$ (4)

where, *d*(*i*,j) is the distance between node *i* and node *j*, i.e., the minimum length of any path connecting node *i* and node *j*. The length of a path is the sum of the weights of its edges.

**Betweenness centrality** measures the intermediary ability of the nodes to act as a medium in the network. In the international trade network of industrial robots, the betweenness centrality is the frequency that a country stands on the shortest path between two other countries. The betweenness centrality of node *i* is given by Eq (5):

$$BC(i)=\sum_{x\neq i\neq y}\frac{\sigma_{xy}(i)}{\sigma_{xy}}$$ (5)

where *σ*_*xy*_ is the total number of shortest paths from node *x* to node *y* and $\frac{\sigma_{xy}(i)}{\sigma_{xy}}$ is the number of these paths that pass through node *i*.

**The block model** is a method and technique that can detect network agglomeration, core-edge structures and hierarchies. Structural equivalence refers to a network in which the entire network structure is not changed if two nodes are replaced by each other. In such as case, the two nodes are equivalent in the network structure, which is also called equivalent class. Structural equivalence is based on the degree to which the rows and columns of nodes are similar. That is, the dissimilarity index between two nodes is the number of other nodes that are not shared by two nodes. After normalization, the value ranges from 0 (completely similar) to 1 (completely different). A higher nonsimilarity value indicates more internal dissimilarity.

If a network contains equivalent class, the block model matrix calculation can be used to classify network nodes; the network with equivalent class is represented by the adjacency matrix, the block in the adjacency matrix form a full or empty network adjacency matrix graph. Therefore, we can simplify the original adjacency matrix into a new adjacency matrix, shrink each original category into a new node, and then label the source type for each category in the new matrix. That is, the block in the new matrix is either full type (expressed by com, referring to the existence of equivalent connection) or none type (indicated by null or hyphen—, meaning there is no equivalent connection). This kind of contraction matrix is also called a shadow matrix [32]. Therefore, the block model contains a partition file and a shadow matrix. The partition file assigns nodes to each equivalent class and divides the adjacency matrix of the network into different blocks. The shadow matrix indicates the relationship types within categories. In summary, the block model describes the overall structure of the network and explains the locations and relationships of each node in the complex network structure.

## Spatial-temporal differentiation of the global industrial robot trade

### Density of the industrial robot trade network

This paper uses the UCINET software to portray the global industrial robot trading network from 1998 to 2017 (S1 File) and to measure the density and average degree of the global industrial robot trading network in different years, as shown in Table 1. From 1998 to 2017, the density, average degree and the total number of ties of the global industrial robot trade network show steady upward trends, which means that there are increasingly more participating countries in the trade and the trade links are getting increasingly closer. From 2015–2017, the network density and average degree tend to be stable, the main producing and consuming countries of industrial robots have relatively stable complementary relationships with respect to their factor endowments, and the global industrial robot trade relationships tend to be stable.

**Table 1:** Network density, average degree and number of ties in the global industrial robot trade.

	**1998**	**1999**	**2000**	**2001**	**2002**	**2003**	**2004**	**2005**	**2006**	**2007**
**Density**	0.443	0.454	0.518	0.515	0.531	0.541	0.551	0.568	0.580	0.595
**Avg Degree**	25.27	25.89	29.552	29.345	30.276	30.828	31.397	32.362	33.052	33.914
**No. of Ties**	1466	1502	1714	1702	1756	1788	1821	1877	1917	1967
	**2008**	**2009**	**2010**	**2011**	**2012**	**2013**	**2014**	**2015**	**2016**	**2017**
**Density**	0.590	0.606	0.615	0.627	0.638	0.641	0.647	0.653	0.655	0.653
**Avg Degree**	33.621	34.517	35.052	35.741	36.345	36.552	36.897	37.224	37.345	37.241
**No. of Ties**	1950	2002	2033	2073	2108	2120	2140	2159	2166	2160

### Centrality of the industrial robot trade network

#### Degree centrality

The larger the centrality of a country is in the global industrial robot trade network, the more countries that have industrial robot trade links with that country, and the more important its trade status. This paper divides the study intervals into four stages, with 1998, 2005, 2012, 2017 as the representatives of the four stages, and the time interval of each stage was 8 or 6 years. In addition, in 2008 and 2009, the global economy was affected by the financial crisis and slightly fluctuated, which we do not consider them as typical sample years. UCINET software is used to calculate the in-degree centralities and out-degree centralities of countries in 1998, 2005, 2012 and 2017 and further select the top 10 countries in different years to depict the evolutionary characteristics of the industrial robot trade status, as shown in Table 2.

**Table 2:** In-degree centrality and out-degree centrality of the global industrial robot trade network.

Out-degree centrality								In-degree centrality							
1998		2005		2012		2017		1998		2005		2012		2017	
Node	Value	Node	Value	Node	Value	Node	Value	Node	Value	Node	Value	Node	Value	Node	Value
JPN	54	JPN	56	DEU	57	DEU	57	DEU	45	DEU	50	DEU	50	NLD	55
DEU	53	CHN	56	CHN	57	CHN	57	GBR	42	ITA	44	FRA	48	DEU	49
FRA	52	DEU	55	ITA	56	NLD	57	ITA	41	USA	44	ITA	47	USA	49
GBR	52	ITA	55	USA	56	AUT	57	USA	40	GBR	43	USA	47	ITA	47
ITA	51	USA	55	CHE	56	ITA	56	SWE	37	FRA	43	GBR	46	FRA	47
CHE	51	CHE	54	CAN	56	ESP	56	NLD	36	CAN	43	CHE	45	CHN	46
CHN	51	BEL	53	KOR	56	USA	55	FRA	34	AUT	42	NLD	45	ESP	46
SWE	50	GBR	53	TUR	56	FRA	55	CHE	34	ESP	42	IND	44	KOR	45
USA	50	FRA	53	FRA	55	KOR	55	AUT	33	CHE	40	AUT	43	AUS	45
NLD	50	AUT	53	AUT	55	BEL	55	MEX	34	KOR	40	CHN	43	GBR	44

Firstly, from the perspective of the in-degree centrality, the import partners of the top ten countries increased in general, ranging from 33 to 55. However, Germany, Italy, Britain and the United States have large numbers of import partners with stable relations. For example, from 1998 to 2017, import partners in Germany, Italy and the UK changed no more than six and all above forty, these countries are at the center of the import trade of industrial robots. The import partners of the Netherlands, South Korea and China have increased significantly. This indicates that the industrial robot industry is developing rapidly. For example, from 1998 to 2017, import partners in Netherlands, South Korea and China increased no less than sixteen. The rapid development of China's industrial robot import trade is attributed to the improvement of its manufacturing industry and the demand for intelligent transformation. Although China's industrial robot production started late, it is quickly embedded into the global value chain. In general, the promotion of intelligent manufacturing transformation and the division of labor in the global industrial robot value chain make the industrial robot import market increasingly diversified and the import trade market structure increasingly dispersed.

Secondly, from the perspective of the out-degree centrality, the export partners of the top ten countries are relatively stable, ranging from 50 to 57,especially Japan, Germany and Italy who have large numbers of export partners with stable relations, and these countries are at the center of the industrial robot trade. The export partners of China, the United States, the Netherlands, and South Korea have increased slightly. In general, the number of trading partners of major industrial robot exporting countries in the world has increased steadily and slightly, while the number of cooperating countries in the export trade is relatively concentrated, and the market structure of export trade is relatively tight.

Thirdly, with respect to the change in the in-degree and out-degree centrality in the same year, global industrial robots show the feature of enhanced intra-industry trade. For example, from 1998 to 2017, Germany and Italy were both core importers and core exporters. In 2017, in addition to Germany and Italy, the Netherlands, China, the United States and France were all important import and export countries, which means that the cooperation of the whole industrial chain of the global industrial robot trade is emerging.

Finally, combined with the analysis of the annual total import and export trade volume of each country, it is found that Japan, Germany, Italy, Sweden and other countries are mainly export-oriented, and the export trade volume is far greater than the import trade volume. For example, from 2013 to 2017, the import trade of industrial robots in Japan accounted for only about 13% of its export trade, while the import trade of industrial robots in Germany accounted for 32% of its export trade. Italy was about 32.6%, and Sweden was 27.4%. In contrast, China, Korea, India, Thailand and other industrial robots ‘catch-up’ countries are mainly import-oriented, and exports of industrial robot products are insufficient. For example, from 2000 to 2017, China's industrial robot export trade only accounted for 35.3% of its import trade, South Korea's industrial robot export trade accounted for 64.8% of import trade, India was about 12.5%, and Thailand was about 13.4%.” In other words, counties like Japan, Germany and Italy still hold the core position in industrial robot trade network.

#### Closeness centrality

The closeness centrality index reflects to what degree a country stands in the central position of the network. The more central a country is, the lower its total distance from all the other nodes. UCINET software is used to calculate the in-closeness centrality and out-closeness centrality of the countries in 1998, 2005, 2012 and 2017, closeness centrality is normalized, and the top 10 countries in different years are shown in Table 3.

**Table 3:** In-closeness centrality and out-closeness centrality of the global industrial robot trade network.

Out-closeness centrality								In-closeness centrality							
1998		2005		2012		2017		1998		2005		2012		2017	
Node	Value	Node	Value	Node	Value	Node	Value	Node	Value	Node	Value	Node	Value	Node	Value
JPN	0.891	CHN	0.983	DEU	1.00	DEU	1.00	DEU	0.722	DEU	0.835	DEU	0.891	NLD	0.950
DEU	0.877	JPN	0.983	CHN	1.00	NLD	1.00	GBR	0.695	ITA	0.770	FRA	0.864	DEU	0.864
FRA	0.864	DEU	0.966	ITA	0.983	CHN	1.00	ITA	0.687	USA	0.770	ITA	0.851	USA	0.864
GBR	0.864	ITA	0.966	USA	0.983	AUT	1.00	USA	0.679	GBR	0.760	USA	0.851	ITA	0.838
ITA	0.851	USA	0.966	CHE	0.983	ITA	0.983	SWE	0.655	FRA	0.760	GBR	0.838	FRA	0.838
CHN	0.851	CHE	0.950	KOR	0.983	ESP	0.983	NLD	0.640	CAN	0.760	CHE	0.826	CHN	0.826
CHE	0.851	GBR	0.934	CAN	0.983	USA	0.966	FRA	0.633	AUT	0.750	NLD	0.826	ESP	0.826
SWE	0.838	FRA	0.934	TUR	0.983	FRA	0.966	CHE	0.626	ESP	0.750	IND	0.814	KOR	0.814
USA	0.838	AUT	0.934	FRA	0.966	KOR	0.966	AUT	0.626	CHE	0.731	CHN	0.803	AUS	0.814
NLD	0.838	BEL	0.934	AUT	0.966	BEL	0.966	MEX	0.626	KOR	0.731	AUT	0.803	GBR	0.803

The results in Table 3 show the following. From the perspective of the out-closeness centrality, before 2005, Japan, Germany and Italy were the core exporting countries of industrial robots. After 2005, China and the Netherlands developed rapidly and gradually became the main core exporting countries. From the perspective of the in-closeness centrality, before 2012, there was little change in the global industrial robot importing countries, which were mainly Germany, Italy, the United Kingdom, and the United States, which have strong industrial robot industry foundations. After 2012, the Netherlands, India, China, South Korea and other countries developed rapidly and became the core importers of the global industrial robot trade. These results are consistent with the conclusions that were reached above.

#### Betweenness centrality

Betweenness centrality characterizes the ability of a network node to control trade resources. The higher the centrality of a node is, the more important the node. Table 4 shows the calculation result of the betweenness centrality. From 1998 to 2017, the overall centrality declined, the participants in the global industrial robot trade became more ‘diversified’, and the gap between the major countries shrank. Germany and Japan have always been at the center of the industrial robot trade. However, in recent years, China, South Korea and the Netherlands have gradually become the core countries due to their increased support for the industrial robot industry. In addition, the trade status of industrial robots in Canada, Turkey and Thailand also improved significantly.

**Table 4:** Betweenness centrality of the global industrial robot trade network.

1998		2005		2012		2017	
Node	nBetweenness	Node	nBetweenness	Node	nBetweenness	Node	nBetweenness
CHE	3.165	JPN	2.033	DEU	1.152	CHN	1.278
JPN	2.830	CHN	2.033	CHN	1.152	AUT	1.278
CHN	2.526	USA	1.829	USA	1.062	DEU	1.278
ESP	2.321	KOR	1.617	KOR	0.989	NLD	1.278
DEU	2.220	ITA	1.563	AUT	0.964	CZE	1.114
FRA	2.166	DEU	1.563	CHE	0.964	KOR	0.975
GBR	1.823	BEL	1.190	TUR	0.964	ITA	0.883
SWE	1.780	FRA	1.149	ITA	0.936	ESP	0.883
USA	1.703	CHE	1.082	CAN	0.936	BEL	0.822
NLD	1.547	AUT	1.076	JPN	0.920	USA	0.812

**Fig 2:**
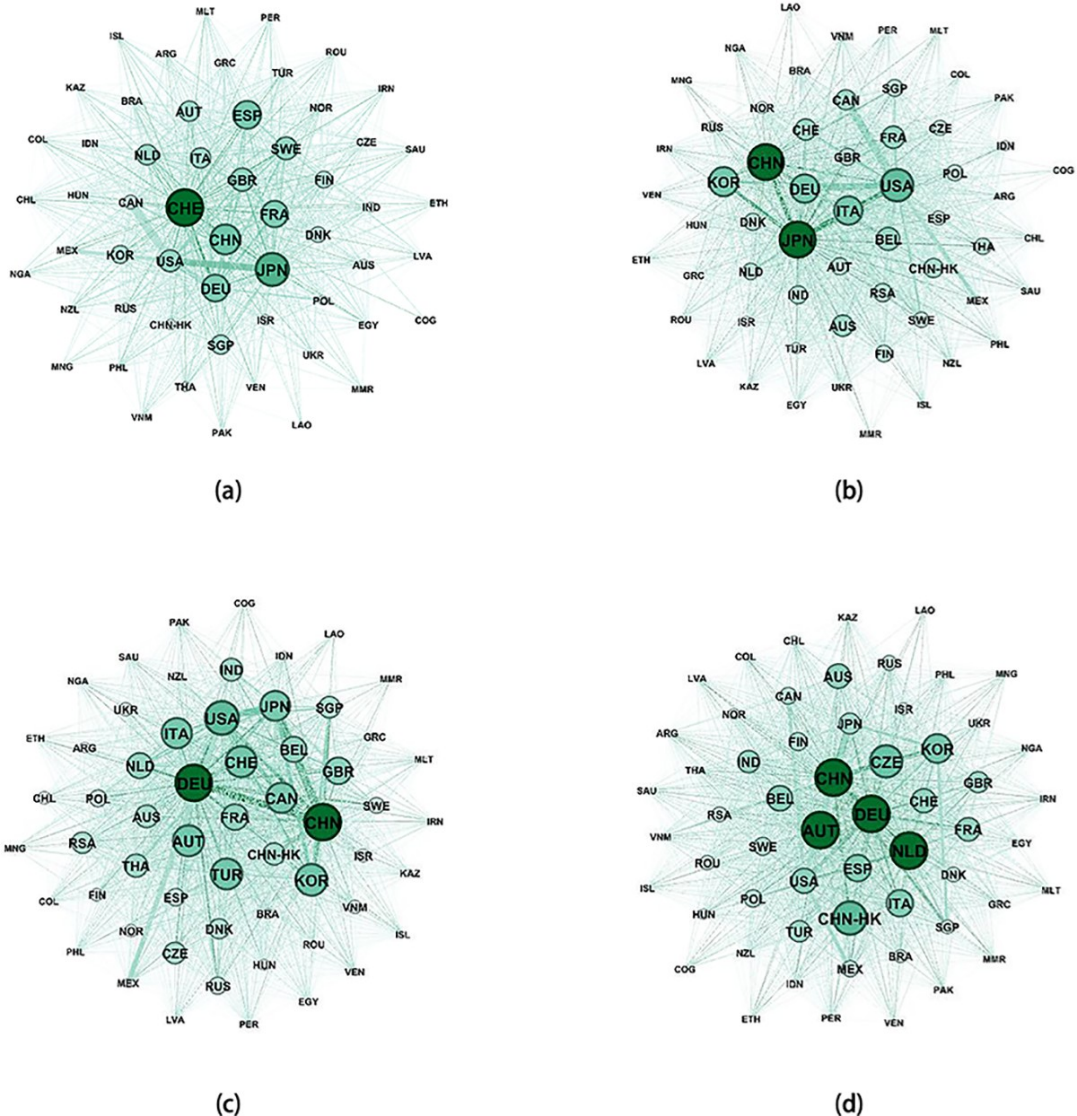
Global industrial robot trade networks in 1998, 2005, 2012 and 2017. Panel(a) shows global industrial robot trade network of 1998. Panel(b) shows global industrial robot trade network of 2005. Panel(c) shows global industrial robot trade network of 2012. Panel(d) shows global industrial robot trade network of 2017.

To better reflect the evolutionary characteristics of the global industrial robot trading network from 1998 to 2017, this paper uses Gephi software to map the network evolution of the global industrial robot trade in 1998, 2005, 2012 and 2017, as shown in Fig 2(A), Fig 2(B), Fig 2(C), and Fig 2(D). Here, a node is ranked by betweenness centrality. Thus, the larger the node is, the greater the betweenness centrality, and the stronger the trade influence.

As shown in Fig 2, in 1998, Switzerland, Japan, Germany, Spain, China, and France were the major industrial robot trading countries. In 2005, Japan and China occupied important positions in the industrial robot trade and were followed by the United States, South Korea, Italy, Germany and other countries. In 2012, Germany and China occupied important positions in the global import and export trade of industrial robots. They had the greatest influences in the global industrial robot trading network and were followed by the United States, South Korea, Austria, Switzerland and other countries. In 2017, the global trade pattern changed slightly, and the trade status of industrial robots in the Netherlands increased rapidly. The most influential node countries in the trade network are China, Austria, Germany and the Netherlands, and they are followed by South Korea, Italy, and the United States.

In addition, the betweenness centrality of the core countries in the world showed a downward trend year by year from 1998 to 2017, which indicates that the differences in the trade statuses are shrinking, and the industrial robot trade is more diversified. Further, by observing the evolutionary trend from 1998 to 2017, it is found that the trade network is increasingly more complex, and there are increasingly more core nodes. It vividly depicts the characteristics of the complexity and diversification of the global industrial robot trade.

## Dynamic evolution of the global industrial robot trade based on the block model

### Dynamic analysis of ‘core-edge’ structure in the global industrial robot trade

To more clearly describe the dynamic evolution of the global industrial robot trade status, this paper analyzes the locations of industrial robot trading countries using complex networks based on the block model. Specifically, UCINET software is used to calculate the ‘K-cores’ of the network. Participating countries are divided using a ‘core-edge’ structure, and the dynamic of ‘core-edge’ structure of the global industrial robot trading network in 1998, 2005, 2012 and 2017 are obtained, as shown in Table 5.

**Table 5:** The ‘core-edge’ regions of the global industrial robot trade network.

	Core countries	Strong semi-peripheral countries	Weak semi-peripheral countries	Peripheral countries
1998	20 (DEU, SWE, CAN, FRA, NLD, CHE, AUT, ITA, USA, GBR, CHN, JPN, KOR, SGP, FIN, NOR, ESP, BRA, ISR)	14 (CZE, HUN, POL, GRC, IND, ROU, UKR, TUR, RUS, AUS, CHN-HK, NZL, PHL, THA)	18 (ETH, PAK, NGA, ISL, MLT, KAZ, LVA, BEL, COG, IRN, SAU, VNM, EGY, IDN, MNG, MMR, LAO, RSA)	6 (ARG, CHL, MEX, COL, VEN, PER)
2005	27 (FRA, CHE, AUT, DEU, ITA, CAN, USA, BEL, DNK, ESP, SWE, GBR, NLD, CHN, JPN, CHN-HK, AUS, KOR, IND, SGP, POL, TUR, CZE, FIN, NOR, HUN, ISR)	7 (IDN, THA, NZL, RSA, ARG, MEX, BRA)	20 (EGY, SAU, IRN, PAK, PHL, VNM, MLT, NGA, ETH, ISL, KAZ, LVA, GRC, ROU, RUS, UKR, PER, VEN, CHL, COL)	4 (COG, MNG, LAO, MMR)
2012	24 (FRA, ITA, CHE, DEU, NLD, USA, AUT, CAN, CHN-HK, IND, ESP, GBR, SWE, AUS, CHN, KOR, JPN, THA, SGP, CZE, DNK, BEL, POL, TUR)	16 (RSA, VNM, BRA, MEX, ARG, NZL, IDN, PHL, FIN, HUN, ROU, ISR, NOR, RUS, GRC, UKR)	10 (COL, PER, VEN, CHL, SAU, KAZ, PAK, EGY, ISL, LVA)	8 (COG, ETH, IRN, MLT, NGA, LAO, MNG, MMR)
2017	28 (IND, CHE, CHN, TUR, CHN-HK, JPN, SWE, SGP, CZE, GBR, AUT, KOR, CAN, DNK, MEX, FIN, AUS, BRA, ISR, POL, NOR, DEU, ESP, USA, NLD, FRA, ITA, BEL)	11 (THA, VNM, NZL, PHL, IDN, RSA, HUN, ROU, GRC, RUS, UKR)	15 (IRN, MLT, MMR, ISL, PAK, EGY, SAU, NGA, KAZ, LVA, LAO, MNG, VEN, COG, ETH)	4 (ARG, COL, PER, CHL)

The partitioning results indicate the following.

Firstly, from 1998 to 2017, the number of core countries in the industrial robot trade has gradually increased and stabilized, and the trade development has been characterized as ‘diversified’. It is worth mentioning that Mexico has grown from a periphery country in 1998 to a core country in 2017, thus becoming the fastest growing country in the world. In addition, the trade statuses of India, Turkey and Australia have also increased significantly.

Secondly, from the perspective of the spatial layout characteristics, the core countries of the global industrial robot trade are concentrated in Europe, East Asia and North America. The South American countries represented by Argentina, Peru, Chile, and Colombia lacked competitive advantages and became peripheral countries in the industrial robot trade. The spatial layout of the strong semi-peripheral countries and the weak semi-peripheral countries is more dispersed.

Thirdly, the numbers of strong semi-peripheral countries and weak semi-peripheral countries decreased slightly between 1998 and 2017, which means that the irreplaceable links between the core countries and peripheral countries are strengthened. The number of strong semi-peripheral countries shows an inverted N-type change, and the number of weak semi-peripheral countries shows an N-type change, which indicates a complementary relationship. The number of peripheral countries is small, and the uncompetitive countries have gradually faded out of the global industrial robot trade market.

### Dynamic evolution of the ‘core-edge’ pattern in the global industrial robot trade

To further describe the trade flow among the participating countries of different categories, it is necessary to explore the dynamic relationships within and between the four categories of core-edge structures, that is, the full type (Com, with the similar structure of two-way trade in imports and exports) and the none type (-null, with the one-way connection of imports). In this paper, Pajek's dissimilarity calculation method is used to calculate the final image matrix of the global industrial robot trade. The dynamic evolution of the ‘core-edge’ pattern in the global industrial robot trade are shown in Tables 6–9.

**Table 6:** Final image matrix of the global industrial robot trade in 1998.

	1	2	3	4	Dissimilarity	Dissimilarity of cluster
1 (Core countries)	-	-	-	-	0.26	1.07
2 (Strong semi-peripheral countries)	-	-	-	--	0.58	
3 (Weak semi-peripheral countries)	Com	-	Com	Com	0.35	0.48
4 (Peripheral countries)	-	-	Com	-	0.20	

**Table 7:** Final image matrix of the global industrial robot trade in 2005.

	1	2	3	4	Dissimilarity	Dissimilarity of cluster
1 (Core countries)	Com	-	Com	Com	0.29	0.69
2 (Strong semi-peripheral countries)	-	-	-	-	0.26	
3 (Weak semi-peripheral countries)	-	-	-	-	0.45	0.61
4 (Peripheral countries)	Com	-	-	Com	0.08	

**Table 8:** Final image matrix of the global industrial robot trade in 2012.

	1	2	3	4	Dissimilarity	Dissimilarity of cluster
1 (Core countries)	Com	Com	Com	-	0.17	0.68
2 (Strong semi-peripheral countries)	-	-	Com	-	0.42	
3 (Weak semi-peripheral countries)	Com	Com	Com	Com	0.30	0.42
4 (Peripheral countries)	-	-	-	-	0.20	

**Table 9:** Final image matrix of the global industrial robot trade in 2017.

	1	2	3	4	Dissimilarity	Dissimilarity of cluster
1 (Core countries)	Com	Com	Com	Com	0.21	0.58
2 (Strong semi-peripheral countries)	-	-	-	-	0.24	
3 (Weak semi-peripheral countries)	Com	Com	-	Com	0.32	0.49
4 (Peripheral countries)	-	-	-	-	0.20	

The partitioning results of the final image matrix in Tables 6–9 show the following.

Firstly, on the whole, the dissimilarity of the ‘core’ cluster is higher than that of the ‘periphery’ cluster, which means that the industrial robot trade relationship within the ‘core’ cluster countries is more complex and the intra-trade structure of the core cluster is ‘diverse’. However, the countries in the ‘peripheral’ cluster share more nodes in the industrial robot trade network, and the trade structure of countries in the peripheral cluster is relatively similar. In addition, the dissimilarity within the ‘core’ cluster decreased year by year. From 1998 to 2017, the dissimilarity decreased from 1.07 to 0.58, which was consistent with the conclusion that the trade status differences between the core countries were narrowing, as measured by the betweenness centrality above.

Secondly, in terms of the four categories of the industrial robot trade, the dissimilarity of the core countries is lower than those of strong semi-peripheral countries and the weak semi-peripheral countries, which means that the trade structures are more similar, and that intra-industry trade is larger. The high dissimilarity in strong semi-peripheral countries and weak semi-peripheral countries indicates that the intragroup trade structure is heterogeneous and is mainly one-way trade links. In addition, the dissimilarity of the core cloud is higher than the internal category of ‘core’ countries, which indicates that the core participating countries in the industrial robot trade network coexist, are ‘diverse’ and are at an ‘equilibrium’. Therefore, ‘equilibrium’ means that it will be difficult for other countries to enter the ‘core countries’ in the future since top active countries have stable trade relationships. From the perspective of a complex network, it is difficult for countries to break the current ‘equilibrium’ and push forward to the next new ‘equilibrium’, which need more time and catastrophic change. However, the Sino-US trade war poses a challenge to the balance of global industrial robot trade.

Thirdly, from the perspective of the import and export trends of the global industrial robot trade, the trade relationships between core countries have been strengthened, and they have changed from a simple import relationship to a two-way import and export relationship. In 2017, the core countries have two-way import and export relations with other categories of countries, which means that the intra-industry trade that is centered on the core countries has been strengthened. The trade relations that are related to ‘strong semi-peripheral countries’ and ‘periphery’ countries are characterized by one-way import trade. There is a stable two-way trade relationship between ‘weak semi-peripheral’ countries and ‘core’ countries, ‘strong semi-peripheral’ countries and ‘periphery’ countries, which also indicates that ‘weak semi-peripheral’ countries will have the potential for greater development in the industrial robot industry in the future.

## Conclusions

Industrial robots are an important part of AI development and a necessary means of intelligent transformation in the manufacturing industry. In this context, based on the Uncomtrade database of the import and export trade data of 58 countries in the world from 1998 to 2017, this paper systematically studies the structural characteristics and evolution of the global industrial robot trade network. The main conclusions are as follows. First, since 1998, the density of the global industrial robot trade network has increased gradually and tended to be stable from 2015 to 2017. It became more complicated and more diversified, and major producers and consumers of industrial robots have had relatively stable complementary factor endowment relationships in recent years. Second, in terms of the out-degree centrality, from 1998 to 2017, the global industrial robot export trade market structure is relatively tight. In terms of the in-degree centrality, the global industrial robot import market has become increasingly diversified, import partners in Netherlands, South Korea and China increased significantly, and the import trade market structure has become increasingly decentralized. Third, in terms of the centrality of complex trade networks, the betweenness centrality of the industrial robot trade in the world has shown a downward trend. The trade pattern has been characterized as ‘diversified’, and the gaps between major countries are shrinking. Germany and Japan have always been at the center of the trade. China, South Korea, and the Netherlands are catching up rapidly and gradually becoming the core countries of industrial robot trade. The trade status of industrial robots in Canada, Turkey, Thailand, and Mexico is also significantly improved.

Our findings bear several managerial implications for countries that develop industrial robots.

Firstly, the global industrial robot trade is dominated by developed countries, with Japan, Germany, the United States, and Italy at the center of the trade. In addition, trade status of ‘catch-up’ countries represented by China has rapidly increased recent years. However, ‘catch-up’ countries’ trade relationship is dominated by imports, and exports of industrial robot products are insufficient. Therefore, ‘catch-up’ countries’ can take advantage of regional cooperation, such as China’s OBOR (One Belt One Road) policy, which will significantly be promoted industrial robot trade within the OBOR countries. Secondly, the node similarity implies that the top active countries have stable trade relationships in the global industrial robot field and that it is hard for other countries to break the ‘equilibrium’. Only if they positively participate in global industrial robot trade and achieve key technological breakthroughs will they break their dependence on imports and catch-up to and surpass core countries. These efforts need the support from the government, which should ensure financial policies, promote regional cooperation, and attract more technical talent. In addition, as stated earlier “the Sino-US trade war poses a challenge to global industrial robot trade” which means both China and the United States need to adjust their trade strategies accordingly to prevent the decline in existing trade status.

Several important limitations of our study highlight possible directions for future research. First, we use 58 major industrial robot trading economies’ data to explore the global trade network, and future research can be extended to more countries. Second, although this study investigated the spatial-temporal variation characteristics and evolution of the global industrial robot trade, what factors influence the global industrial robot trade was not discussed in this study, which represents an avenue for future research. Furthermore, new complex network analysis methods can be used in the future to explore in more detail the evolutionary characteristics of global industrial robot trade such as community structure analysis. Finally, this work focuses only on different industrial robot product trades; different categories of industrial robots may have different characteristics. These limitations notwithstanding, it is believed that the findings in this paper offer some noteworthy insights for both academics and practitioners.

## Supporting information

S1 Appendix List of 58 economies (country or region). (DOCX)

S1 File Dataset matrix for 58 economies from 1998 to 2017. (ZIP)
