# Supplementary material for: Spatial-temporal variation characteristics and evolution of the global industrial robot trade: A complex network analysis
Source: PLoS One. 2019 Sep 26;14(9):e0222785. doi: 10.1371/journal.pone.0222785 (PMC6762107; doi:10.1371/journal.pone.0222785)
Supplement: S1 Appendix — (DOCX) [file pone.0222785.s001.docx]

S1 Appendix. List of 58 economies (Country or Region)

**Abbreviation & name**

CHN-China, JPN-Japan, DEU-Germany, ITA-Italy, USA-United states, KOR- Rep. of Korea,GBR-United Kingdom, FRA-France, SWE-Sweden, CHE-Switzerland, AUT-Austria, NLD-Netherlands, ESP-Spain, BEL-Belgium, TUR-Turkey, CZE-Czechia, AUS-Australia, FIN-Finland, CAN-Canada, NOR-Norway, DNK-Denmark, SGP-Singapore, BRA-Brazil, ISR-Israel, HUN-Hungary, POL- Poland, GRC- Greece, IND- India, ROU- Romania, UKR- Ukraine, RUS- Russian Federation, NZL-New Zealand, PHL-Philippines, THA-Thailand, ETH-Ethiopia, PAK-Pakistan, NGA-Nigeria, ISL-Iceland, MLT-Malta, KAZ-Kazakhstan, LVA- Latvia, COG- Dem. Rep. of the Congo, IRN- Iran, CHN-HK- Hong Kong SAR,SAU -Saudi Arabia, VNM-Viet Nam, EGY- Egypt, IDN- Indonesia, MNG- Mongolia, MMR- Myanmar, LAO- Lao People's Dem. Rep., RSA-South Africa, ARG- Argentina, CHL- Chile, MEX- Mexico, COL- Colombia, VEN- Venezuela, PER- Peru.
